# Ischemic Toes after Venous Thromboembolism: A Difficult Differential Diagnosis with Good Response to Combination Therapy—A Case Report

**DOI:** 10.1155/2012/403685

**Published:** 2012-03-25

**Authors:** Mohammad Bagher Owlia, Ahmad Salimzadeh, Gholmhossein Alishiri, Saeed Kargar

**Affiliations:** ^1^School of Medicine, Shahid Sadoughi University of Medical Sciences, Yazd, Iran; ^2^Rheumatology Research Center, Sina Hospital, Tehran University of Medical Sciences, Tehran, Iran; ^3^Faculty of Medicine, Baqiyatollah University of Medical Sciences, Tehran, Iran; ^4^Department of Surgery, Shahid Sadoughi University of Medical Sciences, Yazd, Iran

## Abstract

The obliteration of the arterial vascular system of toes is considered as a potentially catastrophic event in clinical practices. In most instances, the cessation of arterial blood flow heralds a serious underlying pathology. A definite classification of some cases is somehow difficult and subject to diagnostic challenges. The aim of the present case study is to share and discuss potentially complex and multifactorial mechanisms of some acute vascular events. In this report, we deal with a 46-year-old man with a rather gradual-onset ischemia of his toe who responded favorably to a combination of pulsed glucocorticoid and anticoagulation within a week.

## 1. Introduction

 “Blue toe” is due to the occlusion of blood flow in the digital arteries of feet. The obliteration of vascular system is a potentially catastrophic event in clinical practices. In most instances, the cessation of arterial blood flow heralds a serious underlying pathology. Several mechanisms are involved, but the most common causes are acute arterio-arterial embolism, in-site thrombosis, vasospasm, vasculitis, inflammatory thrombosis, and atherosclerosis [1, 2]. The typical features of the mentioned scenarios are diagnosed easily by some golden standards or classification criteria. Definite classifications of some cases are somehow difficult and subject to more discussion. The aim of the present case study is to share and discuss potentially complex and multifactorial mechanisms of some acute vascular events. Herein, we describe a 46-year-old man with a gradual-onset ischemia of his toes who responded favorably to anti-inflammatory agents and anticoagulation within a week.

## 2. Case Report

A 46-year-old male government employee referred to us with complains of painful bluish discoloration of his toes that he had suffered for two months. In his past history, he was chemically injured in Iran-Iraq war in the 1980s without any significant sequella till his reference to us. He was a smoker, 5 packs per year for 5 years, but he had decreased it to occasional smoking in recent years. He had no history of medication or drug use. His problem had started three months earlier with symptoms suggestive of right lower limb deep vein thrombosis documented with ultrasound imaging. His symptoms, complicated with pulmonary manifestations in which ventilation scan showed a high probability for pulmonary thromboembolism (PTE), resulted in his hospitalization a month before his last admission. He was on oral cyclophosphamide 100 mg/day and oral prednisolone 20 mg/day with a diagnosis of undifferentiated necrotizing vasculitis in the former center where he referred to us. In his present illness, he complained of excessive sensitivity to the cold exposure of his extremities and showed features highly suggestive of Raynaund's phenomenon. He had no history of diabetes, hypertension, dyslipidemia, or any other comorbid condition.

**Figure 1:**
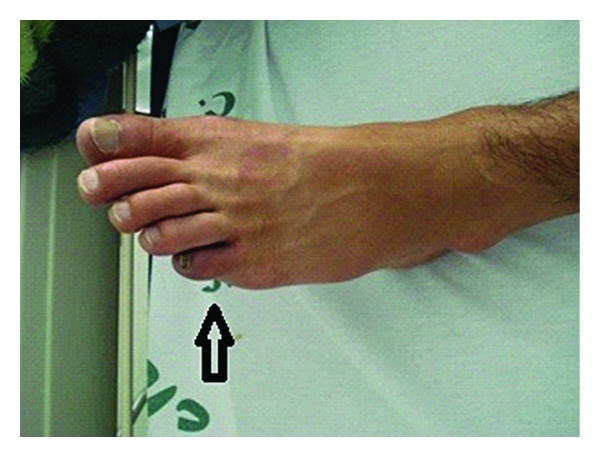
Ischemic discoloration of left small toe two days after treatment.

**Figure 2:**
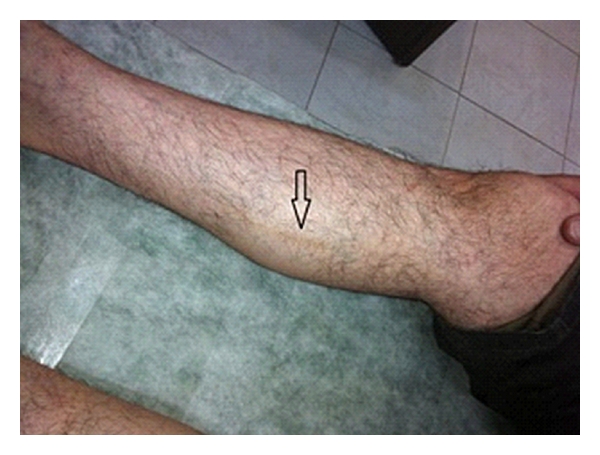
Superficial phlebitis of right shin.

In the physical examination, he showed normal vital signs and also otherwise normal cardiopulmonary exam. In the examination of his extremities, he showed ischemic changes as cold feet with absent dorsalis pedis and posterior tibialis pulses bilaterally along with the ischemic bluish discoloration of small toe at left foot (Figure 1). He also showed typical superficial thrombophlebitis on anteromedial aspect of his right arm and shin (Figure 2). Splinter hemorrhages were evident on some of his finger nails.

In the laboratory investigation, he had a normal blood cell count with normal differentiation. His C3, C4, HBs Antigen, HCV and HIV antibodies, antineutrophil cytoplasmic antibodies (anti MPO and PR3), antinuclear antibodies (Elisa), rheumatoid factor, anti-CCP, anticardiolipin antibodies (Elisa), protein C and S, antithrombin III, factor IV Leiden, prothrombin and thrombin time, partial thromboplastin time, homocysteine, cryoglobulins, cryofibrinogen, and renal and liver function tests were all normal. His CH50 showed some degrees of decreased activity (68%), and the erythrocyte sedimentation rate (Westergren method) was around 24/min on average.

**Figure 3:**
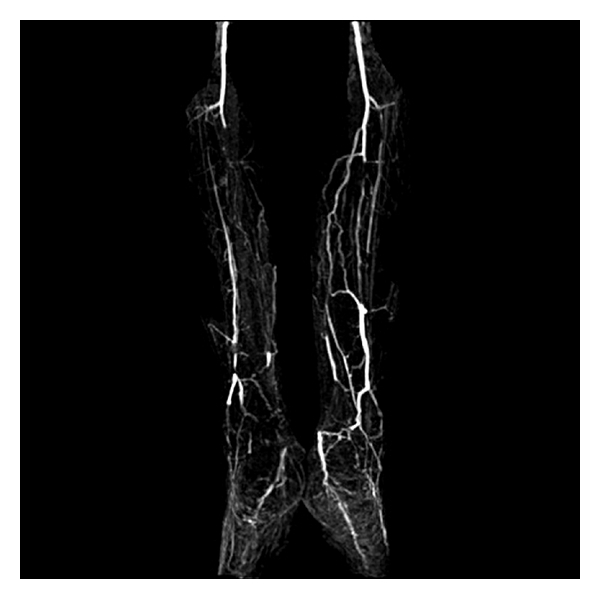
MR angiogram showing bilateral anterior and posterior tibial artery occlusion with some collateral vessels.

His D-dimer assay showed an increase three times above normal. His transthoracic and transesophageal echocardiography had normal results. Three consecutive blood cultures were negative for growing bacteria. Magnetic resonance angiography (MRA) was consistent with bilateral anterior and posterior tibialis artery occlusion (Figure 3).

We had no definite diagnosis at the time of establishing treatment, but we knew that we had to take any useful and safe action to minimize ischemic insult and prevent progression of ischemia. With an assumption of the possible role of a vascular inflammatory disease in setting a hypercoagulable state or an unusual presentation of thromboangiitis obliterans or a combination pathology, we started to administer pulsed intravenous methylprednisolone 1000 mg in three days, pulsed intravenous cyclophosphamide 500 mg at once, pentoxyfylline 400 mg three times a day, aspirin 100 mg/day, heparin 1000 mg/hour for seven days overlapping with oral warfarin 5 mg/day, amlodipine 5 mg daily, and oral daily dose of prednisolone 50 mg/day with rapid tapering to 2.5 mg/day within three weeks after the institution of the mentioned regimen. A rapid clinical improvement with rewarming and reperfusion ensued 5–7 days later and Splinter hemorrhages vanished about a month after treatment. After six months of therapy, while he was on 5 mg/day warfarin, 5 mg/every other day prednisolone, aspirin 81 mg/day and oral cyclophosphamide 100 mg/week and mesna 50 mg/week, there was no complaint of pain and no clinical cyanosis, but his arterial pulses were still nonpalpable. His general condition and function are good now.

## 3. Discussion

Classic “blue toe syndrome” usually refers to a cyanotic single toe most probably due to atheroembolic phenomena with a patent proximal artery. However, the clinical spectrum, differential diagnosis, and etiologies are wide. Vasospasm, vasculitis, hyperviscosity syndrome (cold agglutinin anemia), hypercoagulable state, embolism, calciphylaxis, and toxins (ergot derivatives) are among the most common differential diagnoses. Glucocorticoids and some neoplasms [3] are among the differential diagnoses of blue toe syndrome.

 In typical clinical scenarios (like vascular surgical manipulation of aorta, or index vasoactive drug, typical clinical along with supporting serologic markers), a single mechanism could be assumed. But in several clinical conditions, an interaction of multiple factors may play a role in critical ischemia in toes. In the above-mentioned case, ischemic discoloration of toes after a thrombotic event aroused the suspicion of hypercoagulable state that involves arteries. But a bilateral extensive medium-sized arterial occlusion on MRA made in-situ thrombosis and vasculitis unlikely as a sole mechanism. In such settings, warfarin-induced digital ischemia is an important diagnosis [4], but warfarin had stopped probably inadvertently after the disposition form initial hospitalization. So, drug-induced ischemia is less likely too. The MR angiogram denoted a medium-size involvement which was not compatible with the classical and more common variants of small vessel vasculitic syndromes. The patient had no convincing evidence (aneurysm or seropositivity for HBS antigen) to support the diagnosis of polyarteritis nodosa as a vasculitic pathology involving medium-size vessels. Splinter hemorrhage and superficial thrombophlebitis are the characteristic features of hypercoagulable (antiphospholipid antibody) syndrome or thromboangiitis obliterans (TAO), but occasional cigarette smoking and absence of typical angiographic corkscrew pattern are inconsistent with typical TAO. While typical angiographic pattern is seen in around 17% of TA cases, MRA could miss this pattern in respect to conventional contrast enhanced classic angiography due to some technical limitations. TAO can occur in 5 percent without any history of smoking [5]. Multiple thromboembolism of the pulmonary artery has been reported in cases with TAO. Seronegative antiphospholipid is a possible diagnosis covering this clinical scenario [6, 7]. No relationship between chemical war injuries and thrombotic events was found in our search. Atherosclerotic peripheral arterial disease (PAD) is usually seen in elderly people with prominent proximal intimal lesions or a history of diabetes mellitus or dyslipidemia. So in the present case, the diagnosis of atherosclerotic peripheral vascular disease is unacceptable. The gradual onset of clinical course, bilateral occlusion with clear bifurcations, and redistribution to collateral arteries are the strong pieces of evidence against acute thromboembolism. Also, the patient's chronic presentation with rather good collaterals negatively addresses a diagnosis of classic vasculitis in this case. The rapid restoration of clinical ischemia in this case denotes a more easily reversible component of vascular insult which could be a vascular inflammatory condition. This could be the sine qua non of vasculitides or forme frusta of TAO or even an apparently noninflammatory condition such as Raynaud's phenomenon [8].

Treatment of a known background disease could cause a complete to partial improvement in ischemic toes.

We conclude that, in the clinical setting of critical ischemia with an indefinite mechanism, any clinician should consider a cocktail protocol including vasodilators, antiplatelet agent, anticoagulation, and pulsed glucocorticoid with or without vasodilatory prostaglandins (PG) such as PGI2 (Ioprost) or PGE1 (Alprostadil) as a salvage treatment until a definite diagnosis is made.

## References

[1] Nijhof IS, Majoie IML, Dijkhorst-Oei LT, Bousema MT (2007). Blue toe syndrome; a sign of end-arterial occlusion. *Nederlands Tijdschrift voor Geneeskunde*.

[2] Bégon E, Bouilly P, Cheysson E, Cohen P, Bachmeyer C (2010). Isolated blue toe syndrome as the initial sign of wegener granulomatosis. *American Journal of Medicine*.

[3] Heldenberg E, Rabin I, Cheyn D (2011). Epithelioid hemangioendotheliomia as a rare cause of blue toe syndrome. *Journal of Vascular Surgery*.

[4] Varis J, Kuusniemi K, Heiro M, Järveläinen H (2011). Blue toe syndrome—a rare but possible complication of anticoagulant therapy. *Duodecim*.

[5] Puéchal X, Fiessinger J-N (2007). Thromboangiitis obliterans or Buerger's disease: challenges for the rheumatologist. *Rheumatology*.

[6] Rodriguez-Garcia JL, Bertolaccini ML, Cuadrado MJ, Sanna G, Ateka-Barrutia O, Khamashta MA (2012). Clinical manifestations of antiphospholipid syndrome (APS) with and without antiphospholipid antibodies (the so-called 'seronegative APS'). *Annals of the Rheumatic Diseases*.

[7] Iba Ba J, Mipinda JB, Makanga R (2010). Purple extremities in black-skinned patients: blue toe syndrome as presenting sign of antiphospholipid antibody syndrome. *Médecine Tropicale*.

[8] Herrick AL (2011). Contemporary management of raynaud's phenomenon and digital ischaemic complications. *Current Opinion in Rheumatology*.

